# Exploration of the associations between dysphagia, liquid intake restrictions, and comorbidities in acute stroke: a retrospective chart review

**DOI:** 10.3389/fresc.2026.1763404

**Published:** 2026-06-22

**Authors:** Megan E. Crawford, Stacey Reynolds, Tyler Corson, Roy T. Sabo, Virginia Chu, Catriona M. Steele

**Affiliations:** 1 Virginia Commonwealth University—College of Health Professions, Richmond, VA, United States; 2 Bon Secours Mercy Health—St. Mary's Hospital, Richmond, VA, United States; 3 Virginia Commonwealth University—VCU School of Public Health, Richmond, VA, United States; 4 KITE Research Institute—University Health Network, Toronto, ON, Canada; 5 Temerty Faculty of Medicine, Rehabilitation Sciences Institute, University of Toronto, Toronto, ON, Canada; 6 Canada Research Chair in Swallowing and Food Oral Processing, Ottawa, ON, Canada

**Keywords:** aspiration pneumonia, chart review, deglutition disorders (dysphagia), dietary modifications, risk factors, stroke, texture-modified diet, urinary tract infection (UTI)

## Abstract

**Objective:**

To examine relationships between documented dysphagia, liquid intake restrictions, and comorbidities of aspiration pneumonia and urinary tract infection (UTI) during acute stroke hospitalization.

**Design:**

Exploratory retrospective chart review with binary logistic regression analysis.

**Setting:**

Large urban tertiary care hospital system including two Comprehensive Stroke Centers.

**Subjects:**

Adults (*n* = 1,216) admitted with acute ischemic or hemorrhagic stroke between 2021 and 2023.

**Interventions:**

No experimental intervention. Clinical liquid intake restrictions were recorded as none, thickened liquids, or nil per os (NPO) for more than one day.

**Main outcome measures:**

Frequencies and odds ratios for comorbid diagnoses of aspiration pneumonia and UTI given documentation of dysphagia (ICD-10 R13.10), liquid intake restrictions and covariates of age, sex, stroke type, lesion location and lateralization, NIHSS score, length of stay (LOS), smoking, and dentition. Univariate analyses followed by binary logistic regression models for variables with significant univariate associations.

**Results:**

Dysphagia was documented in 31.7 percent of patients. NPO orders were documented in 23.5 percent and thickened liquids recommendations in 3.5 percent. Aspiration pneumonia and UTI occurred in 9.4 percent and 10 percent, respectively. After controlling for non-independence across several variables of interest, the final regression models showed that liquid intake restrictions were significantly associated with both comorbidities. Female sex and age ≥ 60 were also significantly associated with UTI.

**Conclusions:**

Orders for liquid intake restrictions were significantly associated with increased odds of aspiration pneumonia and UTI. The data suggest a need for improved documentation, timely swallow imaging assessments, and prospective research exploring how the timing of diagnoses, dysphagia assessment and liquid intake recommendations impact the observed relationships.

## Introduction

Dysphagia, or difficulty swallowing, is a common and clinically significant complication following stroke, occurring in approximately 1/3 to 2/3 of affected individuals depending on assessment method, timing, and stroke severity (1–3). The presence of dysphagia in the acute phase has been consistently associated with increased risk of dehydration, malnutrition, pneumonia, poor functional recovery, increased length of hospital stays, post-discharge institutionalization, and mortality (3–7). While aspiration pneumonia has been the primary focus of dysphagia-related research for decades, other infections such as urinary tract infection (UTI) are also highly prevalent after stroke. The etiology of post-stroke infection is multifactorial, involving not only aspiration and altered swallowing but also reduced mobility, bladder dysfunction (neurogenic bladder), immunosuppression, and dehydration (8, 9). Rapid and accurate identification of post-stroke dysphagia is essential because early intervention, whether compensatory, rehabilitative, or diet-based, can significantly alter medical outcomes (4, 5). However, the interplay between dysphagia management decisions and infection outcomes remains poorly understood. The goal of this retrospective chart review study was to explore associations between the prevalence of documented dysphagia, dysphagia management decisions, pneumonia and UTI in acute stroke patients.

A primary concern in individuals with dysphagia post stroke is aspiration (the entry of food, liquid or saliva into the airway)**.** The most common clinical interventions intended to reduce aspiration are temporary *nil per os* orders (NPO, nothing by mouth) or recommendations for diet modifications in which thin liquids are replaced with thickened liquids (10–12) (henceforth referred to collectively as “liquid intake restrictions”). The physiological rationale behind recommending thickened liquids is that their slower bolus flow allows more time for achieving airway protection (12, 13). However, it remains unclear whether these interventions are effective for preventing stroke-associated pneumonia (SAP) (2, 14). Some studies actually report higher rates of pneumonia in acute stroke patients who remain NPO and receive nutrition via nasogastric or enteral feeding tubes (15, 16); however, these findings may be confounded by stroke severity (14). To our knowledge, there have been no randomized clinical trials evaluating the efficacy of thick liquids for preventing SAP; however, evidence from a large trial in adults with Parkinson Disease and/or dementia actually found increased rates of pneumonia, dehydration and other negative sequelae in individuals randomized to receiving extremely thickened liquids over a 3-month timeframe (12). Restricting liquid intake through NPO orders or orders for thickened liquids can also reduce overall fluid consumption, and contribute to downstream complications such as malnutrition, dehydration, UTI, renal impairment and prolonged length of stay in acutely hospitalized adults (17–22). Dehydration is an independent predictor of both pneumonia and UTI in hospitalized patients, including those with stroke (3, 6, 18). Perhaps surprisingly, several studies report high frequencies of dehydration in patients with acute stroke and dysphagia, independent of the provision of fluids via parenteral methods or tube feeding (17, 23). Furthermore, in individuals with post-stroke dysphagia, diet restrictions and loss of oral intake are linked to psychological distress, social isolation, and reduced quality of life (24, 25). These issues point to a need to better understand both the positive and negative impacts of recommendations to restrict the oral intake of thin liquids post-stroke to inform optimal clinical practice.

Electronic medical record-based studies are well-suited for understanding real-world patterns of care, practice variations (for example, whether and when swallow imaging assessments are completed), and potential unintended consequences of standard care interventions. Although these studies are frequently limited by methodological challenges such as a reliance on the presence of diagnostic codes in medical charts (2, 3), retrospective chart analyses remain valuable for identifying large-scale trends and generating hypotheses for future prospective research. Accordingly, the specific aims of this study were to:

1) Describe the frequency of documented dysphagia, orders for liquid intake restrictions, aspiration pneumonia and UTI in a sample of adults admitting to acute care hospital due to stroke
2) Examine post-stroke associations between liquid intake restrictions and (a) aspiration pneumonia and (b) UTI post-stroke, while controlling for demographic and clinical covariates.

## Methods

The study followed recommended practices for retrospective electronic medical record-based analyses in neurologic populations (3, 9). Institutional Review Board approval was obtained prior to data extraction.

### Setting and participants

We conducted a review of electronic medical records from a large urban tertiary-care hospital system that includes two Joint Commission-certified Comprehensive Stroke Centers. Records were identified for patients admitted between January 2021 and December 2023 with a primary or secondary diagnosis of acute ischemic or hemorrhagic stroke, confirmed through both ICD-10 codes and radiologic documentation. Records were included for: (a) adults aged 18 years or older with documented acute ischemic or hemorrhagic stroke; who were (b) admitted to the hospital's neurology or stroke service; and who 3) had a hospital stay of at least 24 h. Records were excluded for: (a) individuals with diagnoses of transient ischemic attack or subarachnoid hemorrhage without cerebral infarction; (b) individuals admitted for elective procedures or non-stroke neurologic conditions such as tumor or trauma; and (c) those with incomplete electronic medical record data that prevented coding or analysis.

### Data extraction process

**Figure 1:**
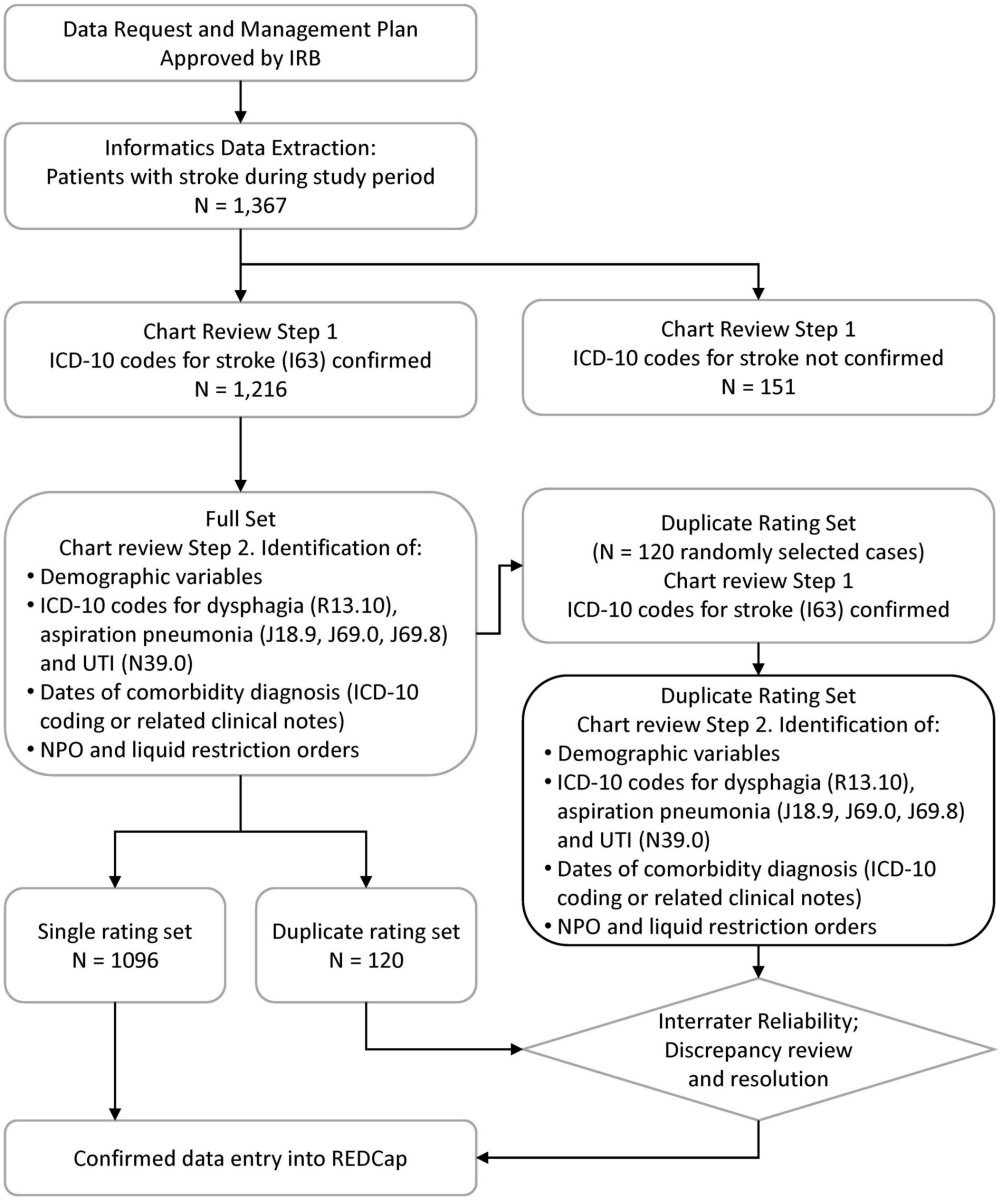
Data Extraction Process. Flow diagram illustrating the data request, initial informatics extraction of all patients with stroke during the study period, verification of ICD-10 stroke codes, extraction of ICD-10 codes for dysphagia, aspiration pneumonia, and urinary tract infection, together with additional clinical variables. Duplicate chart review was performed on a random 10% of the data to assess reliability, with discrepancy review and resolution prior to final confirmed data entry into REDCap.

Figure 1 outlines the chart review and data extraction process. Data extraction was performed by the first author (MEC). Demographic data and dichotomous ratings for the presence/absence of the primary variables of interest were entered directly into a project specific REDCap database (27, 28). Missing data were coded as “unknown”. For the purposes of measuring interrater reliability, duplicate chart review and data extraction were completed for 120 randomly selected records (10% of the sample) by a research assistant blinded to the study purpose and research questions. Agreement between the two raters for dichotomous variables was calculated using Kappa statistics. Prior to further analysis, all personal identifiers were removed in accordance with institutional data-security protocols and Health Insurance Portability and Accountability Act (HIPAA) compliance.

### Variable definitions and coding

#### Primary variables

The four dichotomous primary variables of interest were the presence/absence on ICD-10 diagnostic code for dysphagia, recommendations for liquid intake restrictions, aspiration pneumonia and UTI. Dysphagia was defined as the presence of an ICD-10 diagnostic code for oropharyngeal dysphagia (R13.10) during hospitalization, consistent with previous retrospective electronic medical studies (2, 28). Cases were reviewed to confirm that no prior dysphagia was documented before admission. Liquid-intake recommendations were captured based on the most restrictive diet order documented in the physician's orders and speech-language pathology clinical notes during the admission and categorized as: a) no restrictions (thin liquids permitted); b) thickened liquids [mildly or moderately thick per the International Dysphagia Diet Standardisation Initiative (IDDSI) framework] (29); or c) NPO (no oral intake other than ice chips or sips of water over a time period >24 h, excluding orders related to single-day procedures). Comorbidities were defined by ICD-10 codes for aspiration pneumonia (J69.0, J69.8) and UTI (N39.0) and corroborating chart documentation. Comorbidities were only included if they were documented during the same hospitalization. The timing of comorbidity diagnosis (i.e., number of days post admission) was also extracted.

### Covariates

In addition to the primary variables of interest, information regarding covariates was extracted. These included:

- demographic variables of age (in years); sex assigned at birth; race; insurance status; smoking status and dental status;
- stroke type (ischemic, hemorrhagic or mixed), lesion location (supratentorial, infratentorial or both) and lateralization (left, right or bilateral);
- National Institutes of Health Stroke Scale (NIHSS) score;
- length of stay (LOS);
- documentation of COVID-19 during the admission; and
- documentation of any swallow imaging assessment and type performed during the admission.

### Statistical analysis

The statistical analysis followed a stepwise process:

1) Preliminary analysis of univariate relationships between the two comorbidity outcomes (aspiration pneumonia; UTI) and the independent variables of dysphagia and liquid intake restrictions and all covariates, using cross-tabulations with chi-square tests and odds ratios.
2) Multivariate binary logistic regression models carrying forward significant univariate variables from step 1 to identify variables significantly associated with outcomes of pneumonia and UTI.
3) Model refinement to adjust for non-independence across variables in step 2.

All analyses were conducted using IBM SPSS Statistics, version 30.0 (30) using an alpha criterion of *p* < 0.05. Preliminary Box-Tidwell tests identified violations of the statistical assumption of linearity for the continuous variables of age, NIHSS and LOS. Additionally, due to constraints imposed by the institutional review board for the purposes of protecting privacy, age in years was not available for participants over age 80. Consequently, these variables were dichotomized as follows: (a) age was categorized as <60 vs. ≥60 years, consistent with epidemiologic thresholds used in prior stroke-outcome studies (31, 32); (b) NIHSS scores of stroke severity (3) were dichotomized as <11 and ≥11 based on inspection of the data distribution; and (c) LOS was dichotomized as < vs. ≥11 days based on inspection of the data distribution. Frequencies were calculated for all variables. Further data reduction was performed to ensure sufficient representation of variable subcategories as follows: (a) the Modified Rankin Scale (33) variable was dropped due to >25% missingness; (b) race was dichotomized as White vs. non-White, and also Black vs. non-Black; (c) insurance status was dichotomized as insured vs. uninsured; (d) stroke type, location and lateralization were dichotomized as ischemic vs. hemorrhagic or both, supratentorial vs. infratentorial or both, and left vs. right or bilateral; (e) smoking status was dichotomized as current smoker vs. never or former smoker; (f) dental status was dichotomized as non-denture wearers or non-edentulous participants with at least 5 teeth in the mouth vs. denture wearers or edentulous individuals; (g) swallow imaging was dichotomized as either videofluoroscopic swallowing studies (VFSS) or flexible endoscopic examinations of swallowing (FEES) vs. no imaging; and (h) liquid intake restrictions were dichotomized as none vs. either NPO or thickened liquids orders (combined), with further stratification of the two liquid intake restriction subcategories in the logistic regression models.

## Results

### Interrater reliability

Comparison of data extraction across the two raters on the randomly selected 10% reliability sample showed agreement for variable presence/absence and subcategory coding on 2246/2348 components. Overall, interrater reliability was excellent (96% exact agreement) with a Kappa of 0.913.

### Participant characteristics

From an initial sample of 1,367 records sourced from the informatics department, 1,216 adults met inclusion criteria for analysis based on ICD-10 confirmed stroke diagnosis. The majority had ischemic stroke (82.9%), followed by mixed stroke type (15.5%) and hemorrhagic stroke (1.6%). Mean age was 68 years (SD = 13), and 44.6% were female. Most participants had supratentorial lesions (80.6%), with 37.2% showing right-hemisphere involvement. The median LOS was seven days (interquartile range = 4–10). Table 1 shows frequencies for the primary and covariate variables of interest in the study sample.

**Table 1:** Categorical variables and frequencies (*N* = 1,216).

Variable	Categories	*n* (%)
Sex-assigned-at-birth	Female	542 (44.6)
	Male	674 (55.4)
Age^a^	<60	305 (25.2)
	≥60	911 (74.8)
Length of stay^a^	<11 days	1,018 (83.7)
	≥11 days	198 (16.3)
Race	Black	563 (46.3)
	White	545 (44.8)
	American Indian	5 (0.4)
	Asian	9 (0.7)
	Native Hawaiian	2 (0.2)
	Other/Unknown	92 (7.6)
Insurance	Medicare	749 (61.6)
	Medicaid	249 (20.5)
	Private/Commercial	175 (14.4)
	Uninsured	43 (3.5)
Type of Stroke	Ischemic	1,008 (82.9)
	Hemorrhagic	20 (1.6)
	Both	188 (15.5)
Location of stroke	Supratentorial (Cerebrum and diencephalon)	980 (80.6)
	Infratentorial (Brainstem and/or Cerebellum)	121 (10)
	Both	115 (9.4)
Lateralization of stroke	Left	533 (43.8)
	Right	452 (37.2)
	Bilateral	231 (19.0)
Modified Rankin Scale (MRS)^b^	No significant disability despite symptoms	106 (8.7)
	Slight disability	43 (3.5)
	Moderate disability	49 (4.0)
	Moderately severe disability	60 (4.9)
	Severe disability	7 (0.6)
	Death	1 (0.1)
	Missing	498 (41.0)
Smoking status	Never a smoker	506 (41.6)
	Current smoker	342 (28.1)
	Former smoker	367 (30.2)
	Missing	1 (0.1)
Dental status	Non-denture wearer and non-edentulous (>5 teeth)	934 (76.8)
	Denture wearer	149 (12.3)
	Edentulous	133 (10.9)
NIHSS score on admission	NIHSS <11	813 (66.9)
	NIHSS ≥11	292 (24.0)
	Missing	111 (9.1)
COVID-19^c^	Negative	1,158 (95.2)
	Positive	58 (4.8)
Dysphagia^c^	Absent	831 (68.3)
	Present	385 (31.7)
Swallow imaging assessment	None	1,002 (82.4)
	Videofluoroscopy	192 (15.8)
	Flexible Endoscopy	22 (1.8)
Aspiration Pneumonia^c^	Absent	1,102 (90.6)
	Present	114 (9.4)
Urinary Tract Infection^c^	Absent	1,094 (90)
	Present	122 (10)
Liquid intake restrictions	No liquid restrictions	887 (73)
	Mildly or moderately thick liquids	43 (3.5)
	NPO or oral intake limited to ice chips and/or sips of water	286 (23.5)

a Dichotomized due to violation of statistical assumption in continuous variable form.

b Data collected but ultimately removed from study due to missingness.

c Diagnosis during hospital admission for first acute stroke.

### Dysphagia documentation and swallow imaging assessment

Dysphagia was documented using ICD-10 diagnostic codes in 31.7% of participants (*n* = 385). Swallow imaging assessments were completed for 214 patients (17.6% of the total sample). Table 2 shows details regarding the frequency of swallow imaging assessment provided to the participants with dysphagia. Forty-two percent of these participants underwent VFSS and 4% received a FEES exam.

**Table 2:** Frequencies of instrumental swallowing assessment by dysphagia diagnostic subgroup.

Dysphagia	Swallowing Assessment	*n* (%)
Present (*n* = 385)	None	209 (54)
	VFSS	160 (42)
	FEES	16 (4)
Absent (*n* = 831)	None	793 (95)
	VFSS	32 (4)
	FEES	6 (0.1)

FEES, flexible endoscopic examination of swallowing; VFSS, videofluoroscopy.

### Liquid intake restrictions

Across the entire sample, 73% of patients continued regular oral intake with no restrictions. Among the remaining 27% of the sample, the use of thickened liquids was the most-severe liquid intake restriction recommended for 3.5% of participants, while 23.5% had NPO orders lasting more than 24 h. Additional details regarding the frequency of liquid intake restrictions by dysphagia subgroup can be found in Table 3. Notwithstanding the fact that liquid intake restrictions were recommended for 11% of participants who did not have a documented dysphagia diagnosis, the odds of a dysphagia diagnosis were 11.74-fold higher [95% confidence interval (CI) 8.74 to 15.76] in patients for whom a liquid intake restriction was ordered, suggesting a close correspondence and substantial overlap between these two variables.

**Table 3:** Recommendations for restricted oral intake of liquids by dysphagia diagnostic subgroup.

Dysphagia	No Liquid Restrictions	Thickened Liquids	NPO/Ice Chips Only/Sips of Water Only	Total
	*n (%)*	*n (%)*	*n (%)*	*n*
Present (*n* = 385)	152 (40)	32 (8)	201 (52)	385
Absent (*n* = 831)	735 (89)	11 (1)	85 (10)	831

NPO refers to *nil per os*, or nothing by mouth.

### Comorbidity diagnoses and timing

Aspiration pneumonia occurred in 9.4% of the total sample (*n* = 114) and UTI in 10% (*n* = 122). With regard to timing, average time to aspiration pneumonia diagnosis was five days post admission (median = four days; mode = one day). Average time to UTI diagnosis was four days (median = two days; mode = one day). Overlap between comorbidities was observed, with 18 patients diagnosed with both aspiration pneumonia and UTI during their hospital stay. Among these individuals 12 (67%) also had a diagnosis of dysphagia and 16 (89%) had liquid intake restrictions.

### Univariate relationships

Tables 4, 5 show the results of the univariate analyses, examining associations between independent variables and comorbidity outcomes of aspiration pneumonia and UTI. The presence of a documented ICD-10 code for dysphagia was associated with 3.81-fold higher odds of aspiration pneumonia and 2.56-fold increased odds of UTI. Recommendations for liquid intake restrictions were associated with 23.10-fold increased odds of aspiration pneumonia and 3.32-fold increased odds of UTI. The odds of UTI were significantly higher in participants aged ≥60 years and in females. Covariates of NIHSS ≥11 and LOS ≥11 days were also significantly associated with increased odds of both comorbidities. These two indicators of overall medical severity were also significantly associated with each other, such that an initial NIHSS score ≥ 11 was associated with 5.15-fold higher odds (95% CI: 3.69 to 7.17) of a LOS ≥11 days.

**Table 4:** Frequencies of dichotomized variables of interest in the 114 patients with aspiration pneumonia.

Dichotomized Variable	Absent (*N*)	Absent (%)	Present (*N*)	Present (%)	Pearson Chi-Square	*p*-value (2-sided)	Odds Ratio	95% CI lower bound	95% CI upper bound
Stroke type: Ischemic	41	36.0%	73	64.0%	31.6	<0.001	0.32	0.21	0.48
Age ≥ 60 years	22	19.3%	92	80.7%	2.2	0.135	1.45	0.89	2.35
Stroke lateralization: Right or Bilateral	43	37.7%	71	62.3%	1.9	0.167	1.32	0.89	1.97
Race: Black	71	62.3%	43	37.7%	3.7	0.05	0.68	0.46	1.01
Race: White	57	50.0%	57	50.0%	1.4	0.243	1.26	0.86	1.85
Sex: Female	67	58.8%	47	41.2%	0.6	0.450	0.86	0.58	1.27
ICD-10 Code for dysphagia	45	39.5%	69	**60** **.** **5%**	**48** **.** **4**	**<0** **.** **001**	**3** **.** **81**	**2** **.** **56**	**5** **.** **68**
Current Smoker	87	76.3%	27	23.7%	2.5	0.111	0.70	0.44	1.09
Liquid Restrictions (Binary)	16	14.0%	98	**86** **.** **0%**	**221** **.** **2**	**0** **.** **001**	**23** **.** **10**	**13** **.** **35**	**39** **.** **95**
NIHSS ≥11^a^	34	35.4%	62	**64** **.** **6%**	**78** **.** **7**	**0** **.** **000**	**6** **.** **18**	**3** **.** **96**	**9** **.** **62**
Denture wearer or edentulous	89	78.1%	25	21.9%	0.1	0.738	0.92	0.58	1.47
Stroke location: Infrantentorial	87	76.3%	27	23.7%	1.5	0.225	1.33	0.84	2.10
LOS of 11 days or longer	46	40.4%	68	**59** **.** **6%**	**173** **.** **6**	**0** **.** **000**	**11** **.** **05**	**7** **.** **29**	**16** **.** **76**
Uninsured	113	99.1%	1	0.9%	2.6	0.110	0.22	0.03	1.64
COVID-19	108	94.7%	6	5.3%	0.1	0.795	1.12	0.47	2.67

Significant relationships (*p* < 0.05) are shown in bold.

a Due to missing data the total sample with available NIHSS scores and aspiration pneumonia status was 96.

**Table 5:** Frequencies of dichotomized variables of interest in the 122 patients with UTI.

Dichotomized Variable	Absent (*N*)	Absent (%)	Present (*N*)	Present (%)	Pearson Chi-Square	*p*-value (2-sided)	Odds Ratio	95% CI lower bound	95% CI upper bound
Stroke type: Ischemic	29	23.8%	93	76.2%	4.25	0.039	0.62	0.41	0.98
Age ≥60 years	18	14.8%	104	**85** **.** **2%**	**7** **.** **7**	**0** **.** **006**	**2** **.** **06**	**1** **.** **22**	**3** **.** **45**
Stroke lateralization: Right or Bilateral	50	41.0%	72	59.0%	0.45	0.500	1.14	0.78	1.67
Race: Black	67	54.9%	55	45.1%	0.08	0.780	0.95	0.65	1.38
Race: White	66	54.1%	56	45.9%	0.06	0.800	1.05	0.72	1.53
Sex: Female	40	32.8%	82	**67** **.** **2%**	**28** **.** **1**	**<0** **.** **001**	**2** **.** **83**	**1** **.** **90**	**4** **.** **20**
ICD-10 Code for dysphagia	59	48.4%	63	**51** **.** **6%**	**25** **.** **0**	**<0** **.** **001**	**2** **.** **56**	**1** **.** **75**	**3** **.** **74**
Current Smoker	93	76.2%	29	23.8%	2.7	0.103	0.70	0.45	1.08
Liquid Restrictions (Binary)	59	48.4%	63	**51** **.** **6%**	**41** **.** **5**	**<0** **.** **001**	**3** **.** **32**	**2** **.** **27**	**4** **.** **87**
NIHSS ≥11^a^	70	65.4%	37	**34** **.** **6%**	**4** **.** **1**	**0** **.** **044**	**1** **.** **54**	**1** **.** **01**	**2** **.** **35**
Denture wearer or edentulous	91	74.6%	31	25.4%	0.4	0.540	1.14	0.74	1.76
Stroke location: Infrantentorial	102	83.6%	20	16.4%	0.8	0.375	0.80	0.48	1.32
LOS of 11 days or longer	76	62.3%	46	**37** **.** **7%**	**45** **.** **7**	**0** **.** **00**	**3** **.** **75**	**2** **.** **50**	**5** **.** **62**
Uninsured	121	99.2%	1	0.8%	2.9	0.090	0.21	0.03	1.52
COVID-19	115	94.3%	7	5.7%	0.3	0.597	1.25	0.55	2.81

Significant relationships (*p* < 0.05) are shown in bold.

a Due to missing data the total sample with available NIHSS scores and UTI status was 107 .

### Logistic regression: variables associated with aspiration pneumonia

Table 6 shows the results of the initial logistic regression model for aspiration pneumonia. Variables entered into this model included ICD-10 dysphagia diagnostic code, liquid intake restriction (by type), and NIHSS. At this stage of the analysis, we decided to exclude LOS as a variable, recognizing its significant association with NIHSS and reasoning that it was more likely to be an outcome of rather than a contributing factor to either comorbid outcome. Liquid intake restrictions in the form of thickened liquids or NPO orders were both associated with dramatically increased odds of aspiration pneumonia (10.65– and 34.85-fold, respectively). Neither the documentation of dysphagia as an independent variable nor NIHSS scores ≥11 were significantly associated with increased odds of aspiration pneumonia. Table 7 shows the results of the subsequently refined model, removing dysphagia as an independent variable due both to its suspected overlap with liquid intake restrictions and its non-significance in the previous model iteration, and removing NIHSS based on non-significance. As shown in the first two rows of the table, individuals who either received thickened liquids or NPO orders demonstrated 23-fold higher odds of aspiration pneumonia. This is a strong positive association, although the relatively wide confidence interval suggests some uncertainty regarding the exact magnitude of the relationship. The lower portion of the table separates the effects for thickened liquids vs. NPO orders, showing strong associations between both forms of liquid intake restriction and the odds of aspiration pneumonia.

**Table 6:** Variables associated with aspiration pneumonia (first model).

Predictor	B	SE	Wald	df	*p*	Exp(B)	95% C.I. for EXP(B)	
							LL	UL
Liquid Intake Restrictions			76.31	2	**<0** **.** **001**			
NPO	3.55	0.41	74.96	1	**<0** **.** **001**	34.85	15.60	77.87
Thickened Liquids	2.37	0.65	13.20	1	**<0** **.** **001**	10.65	2.97	38.16
NIHSS ≥ 11	0.34	0.27	1.64	1	0.200	1.40	0.84	2.36
ICD-10 code for Dysphagia	−0.09	0.27	0.12	1	0.730	0.91	0.54	1.54
Constant	−4.51	0.34	174.17	1	**<0** **.** **001**	0.01		

B, beta; SE, standard error; df, degrees of freedom; Exp(B), odds ratio; CI, confidence interval; LL, lower limit; UL, upper limit; NPO, nil per oris; LOS, length of stay; NIHSS, national institutes of health stroke scale.

Significant predictors (*p* < 0.05) are shown in bold font.

**Table 7:** Variables associated with aspiration pneumonia (refined model).

Predictor	B	SE	Wald	df	*p*	Exp(B)	95% C.I. for EXP(B)	
							LL	UL
Liquid Intake Restrictions (combined)	3.14	0.28	126.28	1	**<0** **.** **001**	23.10	13.35	39.95
Constant	−4.0	0.25	251.02	1	**<0** **.** **001**	0.02		
Liquid Intake Restrictions			131.46	2	**<0** **.** **001**			
NPO	3.24	0.28	131.19	1	**<0** **.** **001**	25.40	14.61	44.19
Thickened Liquids	2.36	0.48	23.76	1	**<0** **.** **001**	10.59	4.10	27.34
Constant	−4.00	0.25	251.01	1	**<0** **.** **001**	0.02		

B, beta; SE, standard error; df, degrees of freedom; Exp(B), odds ratio; CI, confidence interval; LL, lower limit; UL, upper limit; NPO, nil per oris; LOS, length of stay; NIHSS, national institutes of health stroke scale.

Significant predictors (*p* < 0.05) are shown in bold font.

### Logistic regression: variables associated with urinary tract infection

Table 8 shows the results of the logistic regression model for UTI. Variables entered into this model included age, sex, ICD-10 dysphagia diagnostic code, liquid intake restriction (by type), and NIHSS. Significantly increased odds of UTI were seen in patients with female sex (2.58-fold), NPO orders (3.18-fold), dysphagia diagnosis (1.8-fold) and age ≥60 years (1.91-fold). The odds of UTI were not significantly impacted by NIHSS scores or recommendations for thickened liquids. In Table 9, the refined model results are shown, excluding dysphagia and NIHSS as independent variables. Whether combined, or treated separately, liquid intake restrictions in the form of thickened liquids or NPO orders were associated with 3-fold higher odds of UTI. Female sex and age ≥60 years also continued to show significant associations with the outcome of UTI, contributing to 2.55-fold and 1.75-fold increased odds, respectively.

**Table 8:** Variables associated with UTI (first model).

Predictor	B	S.E.	Wald	df	Sig.	Exp(B)	95% C.I.for EXP(B)	
							Lower	Upper
Female sex	0.95	0.22	18.05	1	**<0** **.** **001**	2.58	1.67	3.99
Liquid Intake Restrictions			16.95	2	**<0** **.** **001**			
NPO	1.16	0.28	16.74	1	**<0** **.** **001**	3.18	1.83	5.52
Thickened Liquids	0.88	0.51	3.01	1	0.080	2.41	0.89	6.46
ICD-10 code for Dysphagia	0.59	0.25	5.59	1	**0** **.** **020**	1.80	1.11	2.94
Age ≥60 years	0.65	0.30	4.81	1	**0** **.** **028**	1.91	1.07	3.41
NIHSS ≥11	−0.44	0.26	2.76	1	0.097	0.64	0.38	1.08
Constant	−3.83	0.32	140.27	1	**<0** **.** **001**	0.02		

B, beta; SE, standard error; df, degrees of freedom; Exp(B), odds ratio; CI, confidence interval; LL, lower limit; UL, upper limit; NPO, nil per oris; LOS, length of stay; NIHSS, national institutes of health stroke scale.

Significant predictors (*p* < 0.05) are shown in bold font.

**Table 9:** Variables associated with UTI (refined model).

Predictor	B	S.E.	Wald	df	Sig.	Exp(B)	95% C.I.for EXP(B)	
							Lower	Upper
Liquid Intake Restrictions (combined)	1.13	0.20	32.52	1	**<0** **.** **001**	3.08	2.98	4.54
Female sex	0.94	0.21	20.57	1	**<0** **.** **001**	2.55	1.70	3.82
Age ≥ 60 years	0.55	0.27	4.08	1	**0** **.** **043**	1.73	1.02	2.94
Constant	−3.56	0.29	155.43	1	**<0** **.** **001**	0.03		
Liquid Intake Restrictions			33.01	2	**<0** **.** **001**			
NPO	1.14	0.21	31.13	1	**<0** **.** **001**	3.12	2.09	4.66
Thickened Liquids	1.10	0.44	6.07	1	**0** **.** **010**	2.99	1.25	7.14
Female sex	0.94	0.21	20.49	1	**<0** **.** **001**	2.55	1.70	3.83
Age ≥ 60 years	0.56	0.27	4.29	1	**0** **.** **040**	1.75	1.03	2.98
Constant	−3.58	0.29	155.48	1	**<0** **.** **001**	0.03		

B, beta; SE, standard error; df, degrees of freedom; Exp(B), odds ratio; CI, confidence interval; LL, lower limit; UL, upper limit; NPO, nil per oris; LOS, length of stay; NIHSS, national institutes of health stroke scale.

Significant predictors (*p* < 0.05) are shown in bold font.

## Discussion

This exploratory retrospective analysis examined relationships among documented dysphagia diagnosis, liquid intake restrictions, and comorbid infections during acute-stroke hospitalization in a large single-institution cohort. Key study findings can be summarized as follows:

1. ICD-10 diagnostic codes for dysphagia were documented in approximately one third of acute stroke admissions.
2. Less than half of those with documented dysphagia underwent swallow imaging assessment.
3. Nearly one quarter of the total sample was placed on restricted liquid orders. The strong overlap between recommendations for restricted liquid intake and documented dysphagia diagnosis suggests that these two variables were not independent.
4. Approximately one in ten patients developed either aspiration pneumonia and/or UTI during the acute hospital stay.
5. Liquid intake restrictions were strongly associated with dramatically increased odds of aspiration pneumonia.
6. Liquid intake restrictions were also significantly associated with increased odds of UTI, but not with the same magnitude as the relationship observed with aspiration pneumonia

These results illustrate complex relationships between variables and suggest interdependence across some variables such as the documentation of dysphagia and recommendations for liquid intake restrictions. Similarly, prolonged LOS was strongly associated with greater stroke severity as indicated by worse NIHSS scores, suggesting that it may be a consequence of rather than a predictive factor for the comorbidities of interest. Notably, similar observations could be made for Langmore and colleagues' seminal study exploring predictors of aspiration pneumonia in a sample of 189 patients at a Veterans Affairs medical center (34), where initially significant univariate associations between dysphagia and pneumonia faded from significance when additional factors were explored in multivariate analysis. The current study results should be interpreted as showing associations rather than predictive or causative relationships between variables. Notwithstanding this caution, the significant associations seen between liquid intake restrictions and aspiration pneumonia support previous observations that dysphagia is associated with increased risk of respiratory infection in the acute stroke population. Similarly, restricted liquid intake orders that are recommended for stroke patients with dysphagia may be associated with increased risk of UTI. Future studies will need to more carefully control for potential overlap between related independent variables to more clearly elucidate their contributions.

### Liquid restrictions and infection outcomes

Liquid intake restrictions were linked with significantly higher odds of aspiration pneumonia and UTI, even after adjusting for other variables. However, because these infections were frequently documented within the first 48 h of hospitalization, these associations likely reflect clinical decisions to restrict intake among patients who were acutely ill rather than a causal relationship. This interpretation is consistent with prior studies showing that infection can precede or coincide with dysphagia onset rather than result from it (8, 35). Nevertheless, prolonged liquid intake restriction can have unintended consequences. Recommendations for thickened liquids have been shown to reduce overall fluid intake due to decreased palatability and increased feelings of satiety (17). NPO status, when maintained for more than 24 to 48 h, can lead to dehydration, electrolyte imbalances, and impaired mucosal immunity, which may indirectly increase susceptibility to infection (18). Consequently, although thickened liquids are significantly less likely to be aspirated than thin liquids (36), liquid intake restrictions may not prevent aspiration pneumonia or improve overall outcomes (12, 37). Dysphagia management decisions should be individualized, balancing the need for airway protection with the risks of reduced fluid intake and reduced quality of life.

### Swallow imaging assessments

Fewer than half of the patients with a documented dysphagia diagnosis in this study sample underwent swallow imaging assessments. Limited use of or access to VFSS or FEES mirrors national trends in the United States and many other countries and represents an ongoing barrier to evidence-based dysphagia management (2, 28). In many acute-care environments, logistical barriers, clinician availability, and patient instability prevent timely imaging-based evaluation. Consequently, clinicians often rely on bedside findings or precautionary judgment, leading to conservative orders for liquid intake restriction that may persist unnecessarily. Improving access to swallow imaging assessment is critical for confirming the need for and minimizing overuse of liquid intake restrictions. Institutional pathways that promote medically appropriate early swallowing assessment with imaging within the first 24 to 48 h could improve diagnostic precision and optimize care. Similarly, clinicians should monitor hydration and reevaluate liquid intake restriction orders frequently, particularly when recommendations have been based on bedside rather than imaging assessments.

### Comparison with prior literature

The observed infection rates in this study are within the range reported in prior acute stroke cohorts, with aspiration pneumonia affecting approximately 5 to 15% of patients and urinary tract infection affecting 8 to 12% (1, 6, 35). The slightly higher rates observed here likely reflect the inclusion of a medically complex hospital population and a chart review that captured documented infections even when laboratory confirmation was not explicitly stated. In this study, the comorbidities of interest were identified early in hospitalization, suggesting that some cases were either present on admission or developed within the first 48 h of hospitalization, quite possibly prior to formal swallowing evaluation. This temporal pattern is consistent with previous findings that infections may precede, co-occur with or result from dysphagia (8). Other factors, including stroke-related immunosuppression, impaired consciousness, and aspiration during early resuscitation may contribute to cases of aspiration pneumonia. Similarly, prolonged length of hospital stay exposes patients to a greater number of invasive procedures, possibly urinary catheterization, and hospital-acquired pathogens, all of which contribute to heightened infection risk independent of swallowing status (8, 9, 19).

### Limitations

This study has several limitations. Because it relied on electronic medical record data, all variables were contingent on documentation accuracy and coding practices. Dysphagia and infection were identified through diagnostic coding rather than standardized imaging or laboratory criteria, which may have resulted in misclassification. The extracted data did not include information about the methods used to determine presence/absence of dysphagia, nor the timing of dysphagia diagnosis or liquid intake restrictions relative to the documentation of comorbidities. Furthermore, the fact that liquid intake restrictions were recommended for some individuals who did not have dysphagia diagnoses suggests that possibility that dysphagia diagnostic codes were missing in some cases; given that dysphagia is not usually a primary diagnosis, institutional practices around coding may contribute to under documentation of this condition. Finally, in this study, the data regarding liquid intake restrictions reflected the most stringent restriction recorded; it is unknown how many of the individuals for whom an NPO order was in place for longer than 24 h subsequently progressed to thickened liquids.

### Future directions

Future studies should prospectively collect more granular data to enable temporal modeling and validation of the relationships seen in this single center study. In particular, greater detail regarding swallow imaging assessment findings, laboratory test markers of inflammatory processes, immunosuppression and hydration, and time-stamped infection data are needed to better characterize the causal pathways linking dysphagia, liquid intake restriction, and infection. Advanced analytics, including machine-learning models capable of including a larger list of variables, may enhance our understanding of the pathogenesis of post-stroke infections (3, 38).

## Conclusion

This exploratory retrospective study examined relationships between dysphagia, liquid intake restrictions, and comorbidities in adults hospitalized for acute stroke. Across more than one thousand admissions, liquid intake restrictions and markers of overall medical complexity (such as LOS and NIHSS scores) were associated with higher rates of infection. The findings suggest that liquid intake restrictions likely serve as a proxy for dysphagia and greater stroke severity rather than predictors of infection.

## Data Availability

The datasets generated and/or analyzed during the current study are not publicly available due to ethical and legal restrictions. Requests to access the datasets should be directed to Megan Crawford, drmeganecrawford@gmail.com.

## References

[1] Brogan E Langdon C Brookes K Budgeon C Blacker D. Respiratory infections in acute stroke: nasogastric tubes and immobility are stronger predictors than dysphagia. Dysphagia. (2014) 29(3):340–5. 10.1007/s00455-013-9514-5 24445382

[2] Eltringham SA Kilner K Gee M Sage K Bray BD Pownall S. Factors associated with risk of stroke-associated pneumonia in patients with dysphagia: a systematic review. Dysphagia. (2020) 35(5):735–44. 10.1007/s00455-019-10061-6 31493069 PMC7522065

[3] Bond VE Doeltgen S Kleinig TJ Murray J. Dysphagia-related acute stroke complications: a retrospective observational cohort study. J Stroke Cerebrovasc Dis. (2023) 32(6):107123. 10.1016/j.jstrokecerebrovasdis.2023.107123 37058873

[4] Bray BD Smith CJ Cloud GC Enderby P James M Paley L. The association between delays in screening for and assessing dysphagia after acute stroke, and the risk of stroke-associated pneumonia. J Neurol Neurosurg Psychiatry. (2017) 88(1):25–30. 10.1136/jnnp-2016-313356 27298147

[5] Han TS Lean MEJ Fluck D Affley B Gulli G Patel T. Impact of delay in early swallow screening on pneumonia, length of stay in hospital, disability and mortality in acute stroke patients. Eur J Clin Nutr. (2018) 72(11):1548–54. 10.1038/s41430-018-0148-4 29588528

[6] Feng MC Lin YC Chang YH Chen CH Chiang HC Huang LC. The mortality and the risk of aspiration pneumonia related with dysphagia in stroke patients. J Stroke Cerebrovasc Dis. (2019) 28(5):1381–7. 10.1016/j.jstrokecerebrovasdis.2019.02.011 30857927

[7] Martino R Foley N Bhogal S Diamant N Speechley M Teasell R. Dysphagia after stroke: incidence, diagnosis, and pulmonary complications. Stroke. (2005) 36(12):2756–63. 10.1161/01.STR.0000190056.76543.eb 16269630

[8] Poisson SN Johnston SC Josephson SA. Urinary tract infections complicating stroke: mechanisms, consequences, and possible solutions. Stroke. (2010) 41(4):e180–4. 10.1161/STROKEAHA.109.576413 20167905

[9] Boehme AK Kumar AD Dorsey AM Siegler JE Aswani MS Lyerly MJ. Infections present on admission compared with hospital-acquired infections in acute ischemic stroke patients. J Stroke Cerebrovasc Dis. (2013) 22(8):e582–9. 10.1016/j.jstrokecerebrovasdis.2013.07.020 23954599 PMC4782594

[10] Curran J Groher ME. Development and dissemination of an aspiration risk reduction diet. Dysphagia. (1990) 5(1):6–12. 10.1007/BF02407388 2202558

[11] Garcia JM Chambers E 4th, Molander M. Thickened liquids: practice patterns of speech-language pathologists. Am J Speech Lang Pathol. (2005) 14(1):4–13. 10.1044/1058-0360(2005/003) 15962843

[12] Robbins J Gensler G Hind J Logemann JA Lindblad AS Brandt D. Comparison of two interventions for liquid aspiration on pneumonia incidence: a randomized trial. Ann Intern Med. (2008) 148(7):509–18. 10.7326/0003-4819-148-7-200804010-00007 18378947 PMC2364726

[13] Steele CM Bayley MT Bohn MK Higgins V Peladeau-Pigeon M Kulasingam V. Reference values for videofluoroscopic measures of swallowing: an update. J Speech Lang Hear Res. (2023) 66(10):3804–24. 10.1044/2023_JSLHR-23-00246 37669617 PMC10713020

[14] Eltringham SA Kilner K Gee M Sage K Bray BD Pownall S. Impact of dysphagia assessment and management on risk of stroke-associated pneumonia: a systematic review. Cerebrovasc Dis. (2018) 46(3-4):97–105. 10.1159/000492730 30199856

[15] Arnold M Liesirova K Broeg-Morvay A Meisterernst J Schlager M Mono ML. Dysphagia in acute stroke: incidence, burden and impact on clinical outcome. PLoS One. (2016) 11(2):e0148424. 10.1371/journal.pone.0148424 26863627 PMC4749248

[16] Maeshima S Osawa A Miyazaki Y Seki Y Miura C Tazawa Y. Influence of dysphagia on short-term outcome in patients with acute stroke. Am J Phys Med Rehabil. (2011) 90(4):316–20. 10.1097/PHM.0b013e31820b13b2 21765247

[17] Whelan K. Inadequate fluid intakes in dysphagic acute stroke. Clin Nutr. (2001) 20(5):423–8. 10.1054/clnu.2001.0467 11534937

[18] Murray J Scholten I Doeltgen S. Factors contributing to hydration, fluid intake and health status of inpatients with and without dysphagia post stroke. Dysphagia. (2018) 33(5):670–83. 10.1007/s00455-018-9886-7 29497831

[19] Caccialanza R Klersy C Cereda E Bonoldi A Bonardi C Marinelli M. Nutritional parameters associated with prolonged hospital stay among ambulatory adult patients. Can Med Assoc J. (2010) 182(17):1843–9. 10.1503/cmaj.091977 20940233 PMC2988532

[20] Cimoli M Gibney J Lim M Castles J Dammert P. Nil per os in the management of oropharyngeal dysphagia-exploring the unintended consequences. Front Rehabil Sci. (2024) 5:1410023. 10.3389/fresc.2024.1410023 38957683 PMC11217566

[21] Munkholm M Mortensen J. Mucociliary clearance: pathophysiological aspects. Clin Physiol Funct Imaging. (2014) 34(3):171–7. 10.1111/cpf.12085 24119105

[22] Zhang GH Castro R. Role of oral mucosal fluid and electrolyte absorption and secretion in dry mouth. Chin J Dent Res. (2015) 18(3):135–54. http://www.cjdrcsa.com/uploads/media/170207/CJDR-2015-03-1.pdf 26485506

[23] Crary MA Carnaby GD Shabbir Y Miller L Silliman S. Clinical variables associated with hydration status in acute ischemic stroke patients with dysphagia. Dysphagia. (2016) 31(1):60–5. 10.1007/s00455-015-9658-6 26497649

[24] Al Rjoob M Hassan NFHN Aziz MAA Zakaria MN Mustafar MFBM. Quality of life in stroke patients with dysphagia: asystematic review. La Tunisie Medicale. (2022) 100(10):664–9. 36571750 PMC9940652

[25] Hepper EC Wilson J Drinnan M Patterson JM. Psychosocial impacts of being nil-by-mouth as an adult: a scoping review. J Adv Nurs. (2024) 80(9):3499–515. 10.1111/jan.16100 38414146

[26] Harris PA Taylor R Thielke R Payne J Gonzalez N Conde JG. Research electronic data capture (REDCap)-A metadata-driven methodology and workflow process for providing translational research informatics support. J Biomed Inform. (2009) 42(2):377–81. 10.1016/j.jbi.2008.08.010 18929686 PMC2700030

[27] Harris PA Taylor R Minor BL Elliott V Fernandez M O'Neal L. The REDCap consortium: building an international community of software partners. J Biomed Inform. (2019) 95:103208. 10.1016/j.jbi.2019.103208 31078660 PMC7254481

[28] Cohen DL Roffe C Beavan J Blackett B Fairfield CA Hamdy S. Post-stroke dysphagia: a review and design considerations for future trials. Int J Stroke. (2016) 11(4):399–411. 10.1177/1747493016639057 27006423

[29] Cichero JAY Lam P Steele CM Hanson B Chen J Dantas RO. Development of international terminology and definitions for texture-modified foods and thickened fluids used in dysphagia management: the IDDSI framework. Dysphagia. (2017) 32(2):293–314. 10.1007/s00455-016-9758-y 27913916 PMC5380696

[30] IBM Corp. IBM SPSS Statistics for Windows. Version 29.0. Armonk (NY): IBM Corp. (2022).

[31] Li J Zhang P Tao W Dong W Wang Y Xu T. Age-specific clinical characteristics and outcome in patients over 60 years old with large hemispheric infarction. Brain Behav. (2018) 8(12):e01158. 10.1002/brb3.1158 30566281 PMC6305916

[32] Scott CA Li L Rothwell PM. Diverging temporal trends in stroke incidence in younger versus older people: a systematic review and meta-analysis. JAMA Neurol. (2022) 79(10):1036–48. 10.1001/jamaneurol.2022.1520 35943738 PMC9364236

[33] Banks JL Marotta CA. Outcomes validity and reliability of the modified Rankin scale: implications for stroke clinical trials: a literature review and synthesis. Stroke. (2007) 38(3):1091–6. 10.1161/01.STR.0000258355.23810.c6 17272767

[34] Langmore SE Terpenning MS Schork A Chen Y Murray JT Lopatin D. Predictors of aspiration pneumonia: how important is dysphagia? Dysphagia. (1998) 13(2):69–81. 10.1007/PL00009559 9513300

[35] Li YM Xu JH Zhao YX. Predictors of urinary tract infection in acute stroke patients: a cohort study. Medicine. (2020) 99(27):e20952. 10.1097/MD.0000000000020952 32629702 PMC7337551

[36] Borders JC Steele CM. The effect of liquid consistency on penetration-aspiration: a Bayesian analysis of two large datasets. Front Rehabil Sci. (2024) 5:1337971. 10.3389/fresc.2024.1337971 38463609 PMC10920265

[37] Hansen T Beck AM Kjaersgaard A Poulsen I. Second update of a systematic review and evidence-based recommendations on texture modified foods and thickened liquids for adults (above 17 years) with oropharyngeal dysphagia. Clin Nutr ESPEN. (2022) 49:551–5. 10.1016/j.clnesp.2022.03.039 35623866

[38] Kuo YW Huang YC Lee M Lee TH Lee JD. Risk stratification model for post-stroke pneumonia in patients with acute ischemic stroke. Eur J Cardiovasc Nurs. (2020) 19(6):513–20. 10.1177/1474515119889770 31735079

